# O structured reporting, where art thou?

**DOI:** 10.1007/s00330-023-10465-x

**Published:** 2023-11-27

**Authors:** Daniel Pinto dos Santos, Renato Cuocolo, Merel Huisman

**Affiliations:** 1 Department of Diagnostic and Interventional Radiology, Faculty of Medicine and University Hospital Cologne, University of Cologne, Cologne, Germany; 2 Department of Radiology, University Hospital Frankfurt, Frankfurt, Germany; 3 Department of Medicine, Surgery and Dentistry, University of Salerno, Baronissi, Italy; 4 Department of Radiology and Nuclear Medicine, Radboud University Medical Center, Nijmegen, The Netherlands

The opinion paper published in 2014 by Bosmans et al is certainly among the most memorable manuscripts on the topic of structured reporting [1]. In their manuscript the authors compare structured reporting in radiology to the fusion reactor—“an eternal promise, the practical use of which will always be a few decades away.” Interestingly, since the publication of this statement, another decade has gone by and still it seems that structured reporting has not yet found widespread adoption in daily practice. Even though in theory the fusion reactor—as the authors suggest—could be ready and is only “hungry for fuel,” it seems some engineering hurdles and organizational transformation challenges still need overcoming to make the technology work, similar to the actual fusion reactor that as of 2023 has not yet reached sustained net power production [2].

It would be unreasonable to speculate how many more decades radiologists will have to wait until structured reporting will be an integral part of daily practice. But one thing should be clear: not only will it transform how we practice radiology, but also it will unlock new possibilities to interact with the radiological report. However, the same question remains open: how do we get there? Following the paper published in 2018, the European Society of Radiology (ESR) recently published an update on the state of affairs regarding structured reporting in Europe and around the world [3, 4]. This publication might provide some clues to what the next steps could (should?) be. The informal survey conducted as part of this update clearly reveals that many national radiological societies are independently working on collecting structured reporting templates. However, so far (apart from the USA to some extent), no cross-institutional applications of such templates are reported, neither are there any incentives for their usage. From this, two important action items can be deduced. Firstly, given that medicine is more and more evidence based and globalized, forces need to be joined to harmonize structured reporting templates and establish best practices. And secondly, incentives including all necessary resources, need to be provided—at least during a transitory phase—to encourage radiologists in Europe and around the world to adopt structured reporting, and for this policy makers need to be made aware of the potential of structured reporting. If—as many would agree—“data is the new oil,” it would be a waste of valuable resources if the data contained in radiological reports was to be left untapped. In a twist to the analogy of the fusion reactor, in this sense, structured report data itself would be the fuel to power a more data-driven approach to healthcare, where, e.g., the follow-up of incidental nodules could be optimized through automated scheduling [5].

Even though the radiological community at large seems to agree on the benefits of structured reporting, until recently, the most important argument against the widespread adoption of template-based structured reporting remained the disruption to the radiologists traditional speech-based workflow [6, 7]. Given the escalating workloads faced by radiologists, reluctance to alter existing workflows is understandable, especially when no additional time or resources are allocated for the transition. Furthermore, the adoption of a new operational approach often leads to a temporary decrease in reporting speed and a potential increase in error rates. However, with the advent of large language models (LLMs), it could well be that it will not take another decade to solve this very specific engineering challenge [8, 9]. In fact, LLMs have shown to be able to structure unstructured reports and match free-text parts to the respective parts of a structured report template, and possibly even convert text to mineable data formats [10, 11]—another coincidental similarity with fusion power where AI is used to solve the engineering challenge of quickly adjusting the magnetic field to maintain a stable plasma [2]. Consequently, it can be expected that first vendors will be showcasing their LLM-powered reporting solutions in the very near future.

Relying on LLMs or other commercial AI-based solutions to solve the issue of transitioning to structured reporting may also lead to novel challenges in the future. Apart from vendor lock-in, these approaches may become a further economic burden to be shouldered by healthcare systems, many of which are already struggling to meet the demands of current standards of care and technology. In turn, widespread availability of structured reports and leveraging them to train more advanced clinical decision support systems may further widen the gap between the haves and have nots on a global scale and accentuate biases in clinical practice [12]. Alternatively, centers or individual radiologists may start to deploy open-source LLM solutions, which obviously also require some technical support and integration, and may carry the risk of data leakage if deployed without the necessary knowledge.

But even with these challenges hopefully soon solved—as the ESR rightly states in their position statement—it seems that structured reporting is not at the end of its odyssey, yet. Nevertheless, a strong incentive for structured reporting (or a disincentive for free-text reports) will be essential to enable radiologists to invest in these new technologies and help the healthcare system leverage the full potential of the information contained in the radiologists’ reports.

## Declarations

### Guarantor

The scientific guarantor of this publication is PD Dr. Daniel Pinto dos Santos, University Hospital of Cologne (daniel.pinto-dos-santos@uk-koeln.de).

### Statistics and biometry

No complex statistical methods were necessary for this paper.

### Methodology

• commentary
